# Development and implementation of a nurse-based remote patient monitoring program for ambulatory disease management

**DOI:** 10.3389/fdgth.2022.1052408

**Published:** 2022-12-14

**Authors:** Jordan D. Coffey, Laura A. Christopherson, Ryan D. Williams, Shelby R. Gathje, Sarah J. Bell, Dominick F. Pahl, Lukas Manka, R. Nicole Blegen, Michael J. Maniaci, Steve R. Ommen, Tufia C. Haddad

**Affiliations:** ^1^ Center for Digital Health, Mayo Clinic, Rochester, MN, United States; ^2^ Integrity & Compliance Office, Mayo Clinic, Rochester, MN, United States; ^3^ Research Administrative Services, Mayo Clinic, Rochester, MN, United States; ^4^ Department of Nursing, Mayo Clinic, Rochester, MN, United States; ^5^ Division of Hospital Internal Medicine, Mayo Clinic, Jacksonville, FL, United States; ^6^ Department of Cardiology, Mayo Clinic, Rochester, MN, United States; ^7^ Department of Oncology, Mayo Clinic, Rochester, MN, United States

**Keywords:** remote patient monitoring (RPM), ambulatory disease management, digital health, supportive care, telemedicine, chronic disease management, acute disease management

## Abstract

**Introduction:**

Numerous factors are intersecting in healthcare resulting in an increased focus on new tools and methods for managing care in patients' homes. Remote patient monitoring (RPM) is an option to provide care at home and maintain a connection between patients and providers to address ongoing medical issues.

**Methods:**

Mayo Clinic developed a nurse-led RPM program for disease and post-procedural management to improve patient experience, clinical outcomes, and reduce health care utilization by more directly engaging patients in their health care. Enrolled patients are sent a technology package that includes a digital tablet and peripheral devices for the collection of symptoms and vital signs. The data are transmitted from to a hub integrated within the electronic health record. Care team members coordinate patient needs, respond to vital sign alerts, and utilize the data to inform and provide individualized patient assessment, patient education, medication management, goal setting, and clinical care planning.

**Results:**

Since its inception, the RPM program has supported nearly 22,000 patients across 17 programs. Patients who engaged in the COVID-19 RPM program experienced a significantly lower rate of 30-day, all-cause hospitalization (13.7% vs. 18.0%, *P* = 0.01), prolonged hospitalization >7 days (3.5% vs. 6.7%, *P* = 0.001), intensive care unit (ICU) admission (2.3% vs. 4.2%, *P* = 0.01), and mortality (0.5% vs. 1.7%, *P* = 0.01) when compared with those enrolled and unengaged with the technology. Patients with chronic conditions who were monitored with RPM upon hospital discharge were significantly less likely to experience 30-day readmissions (18.2% vs. 23.7%, *P* = 0.03) compared with those unmonitored. Ninety-five percent of patients strongly agreed or agreed they were likely to recommend RPM to a friend or family member.

**Conclusions:**

The Mayo Clinic RPM program has generated positive clinical outcomes and is satisfying for patients. As technology advances, there are greater opportunities to enhance this clinical care model and it should be extended and expanded to support patients across a broader spectrum of needs. This report can serve as a framework for health care organizations to implement and enhance their RPM programs in addition to identifying areas for further evolution and exploration in developing RPM programs of the future.

## Introduction

Numerous factors are intersecting in healthcare resulting in an increased focus on new tools and methods for managing care in patients' homes (1, 2). The need for such programs was accelerated and expanded in response to the Coronavirus Disease 2019 (COVID-19) pandemic to maintain clinical continuity and reduce transmission risk by supporting patient self-management of their medical conditions in their home environment (3–12). In 2019 there were 54.1 million U.S. citizens over the age of 65 (13), by 2030 that number is projected to increase to 74 million (14, 15) with growth of almost 18 million between 2020 and 2030 alone (14, 16). Nearly 60% of adults in the United States have one or more chronic diseases, with the majority experiencing multiple (17). As our aging population increases, so will the number of individuals with chronic diseases, including heart failure, diabetes, hypertension, and respiratory conditions (18, 19). As a result of this increasing chronically ill population combined with limited hospital capacity (20), physician and other healthcare team shortages (21–23), and the impact of the Affordable Care Act (24), many healthcare organizations are developing programs to manage their chronically ill patients (20, 25). Additionally, in recent years, widespread efforts to reduce excess hospital readmissions and length of stay have been spurred by heightened awareness of both the prevalence of readmissions (25, 26) as well as new financial penalties linked to readmission rates and avoidable hospital days (27–30). Further, as there is an increasing movement towards value-based payment models, healthcare organizations are increasingly focused on reducing unnecessary or preventable utilization (31–33).

The high rate of hospital readmissions in the United States (26) has been at the forefront of issues for healthcare leaders since 2009 (34). With the introduction of the Hospital Readmission Reduction Program (HRRP) (27, 28), there has been a gradual decrease in the 30-day readmission rate (29). However, this observation has been controversial as measures under HRRP have been found to be unreliable (35) and potentially associated with an increase in morbidity and mortality (30, 36). Further, there has been evidence that some of the reduction in readmission rates was driven by changes in coding intensity rather than program impact (30, 37).

wInterventions designed to impact patient readmissions can be categorized into three broad domains: pre-discharge interventions (patient education, discharge planning, medication reconciliation, appointment scheduled before discharge); post-discharge interventions (timely follow-up, timely primary care provider communication, follow-up telephone call, patient hotline, home visit); and bridging interventions (transition coach, patient-centered discharge instructions, provider continuity) (25, 38). Overall, programs that are effective in reducing hospital readmissions include those that provide comprehensive, empowering post-discharge support to patients and caregivers and that focus on improved discharge planning, patient education, and follow-up communication (38–40).

Remote patient monitoring (RPM) is an option to provide care at home and maintain a connection between patients and providers to address ongoing medical issues (3, 41). It involves collecting and analyzing patient-generated health data (PGHD) digitally transmitted from the patient to the physician or other qualified health care professional for analysis and management of a treatment plan related to a chronic and/or acute health illness or conditions (41–44). The PGHD may include subjective patient-reported symptom assessments and objective physiologic data, such as weight, blood pressure, oxygen saturation, heart rate, blood glucose, physical activity, sleep patterns, etc. RPM programs support short- and long-term health management goals for primary or specialty care, post-procedural, and acute care (42). The ability to capture PGHD with digital health technologies in near real time increases the capacity for early detection of adverse health trends, early intervention, and rapid response. This early detection may subsequently decrease acute care utilization, including emergency department (ED) visits, hospital admissions and readmissions (42).

## Materials and equipment

Key elements of RPM to be considered include, (1) **Technology framework:** wearables, devices, and apps that can aggregate, analyze, and integrate subjective and objective PGHD with the electronic health record (EHR); (2) **Clinical operational model:** product and operations teams who support implementation of the technology within the clinical practice; use of standardized, evidence-based clinical protocols to program technology monitoring/alerts and interventions for disease management; centralized nursing to provide patient education and health coaching, triage symptoms and adverse data trends, and escalate care to providers as needed; (3) **Technology supply chain and support:** infrastructure to support inventory management, packing, delivery and retrieval of RPM technology kits; kits include the devices and wearables for monitoring, as well as resources to support the usage of devices and patient education materials (4) **Legal, policy, and reimbursement:** legal and regulatory expertise to support development of policies and procedures that ensure proactive compliance with federal and state laws regarding remote clinical care delivery, licensure, and billing.

### Technology framework

RPM technology ranges from apps, wearables, and peripheral medical grade devices to collect, transmit and analyze PGHD. Embedded decision trees and logic drive response to the data, including feedback to the monitored patient and alerts to the centralized nursing team with an overarching goal to help patients meet their personal health goals (42). Software can be used to distribute educational content and videos, enable synchronous telehealth video visits, and administer questionnaires for self-reported medication adherence, nutrition/hydration status, and symptom assessment (44). Advances in mobile and sensor technology, as well as increased adoption of cell phone and tablet use across a broad section of the population, have improved patient adoption and ease of use (44). For billing, physiologic data must be collected by a qualifying “medical device” and “interactive communication” between the provider and patient for a minimum duration *via* audio and/or video platform (27). This patient data collection occurs at a distance, or remotely, from the health care team.

The technology available for use with RPM programs generally has the same functions: to collect and transmit patient-reported symptoms and vital sign data that is collected from medical grade devices (41, 45). The RPM technology kit provides patients with a cellular-enabled smart device (tablet or phone) to avoid issues with broadband access. The smart device is paired with Bluetooth-enabled devices and sensors that collect and analyze data, such as vital signs. The smart device operates like a hub to receive the data transmitted from the peripheral devices, and patient questionnaire responses, which are then transmitted to a secure cloud-hosted platform. These data are integrated into the EHR and made visible to patients through the smart device to provide feedback and engage them in monitoring their health and progress. The RPM software alerts the care team when the patient's data falls outside pre-defined limits. These are configurable at the program and patient level, enabling the care team to monitor patients based on an individual's needs and plan of care. A dashboard available through the software platform allows RPM registered nurses (RNs) to monitor patients. These nurses can view and respond to real-time alerts of patients when data falls outside the pre-defined limits. The dashboard enables the care team to view program and patient-level data.

**Figure 1:**
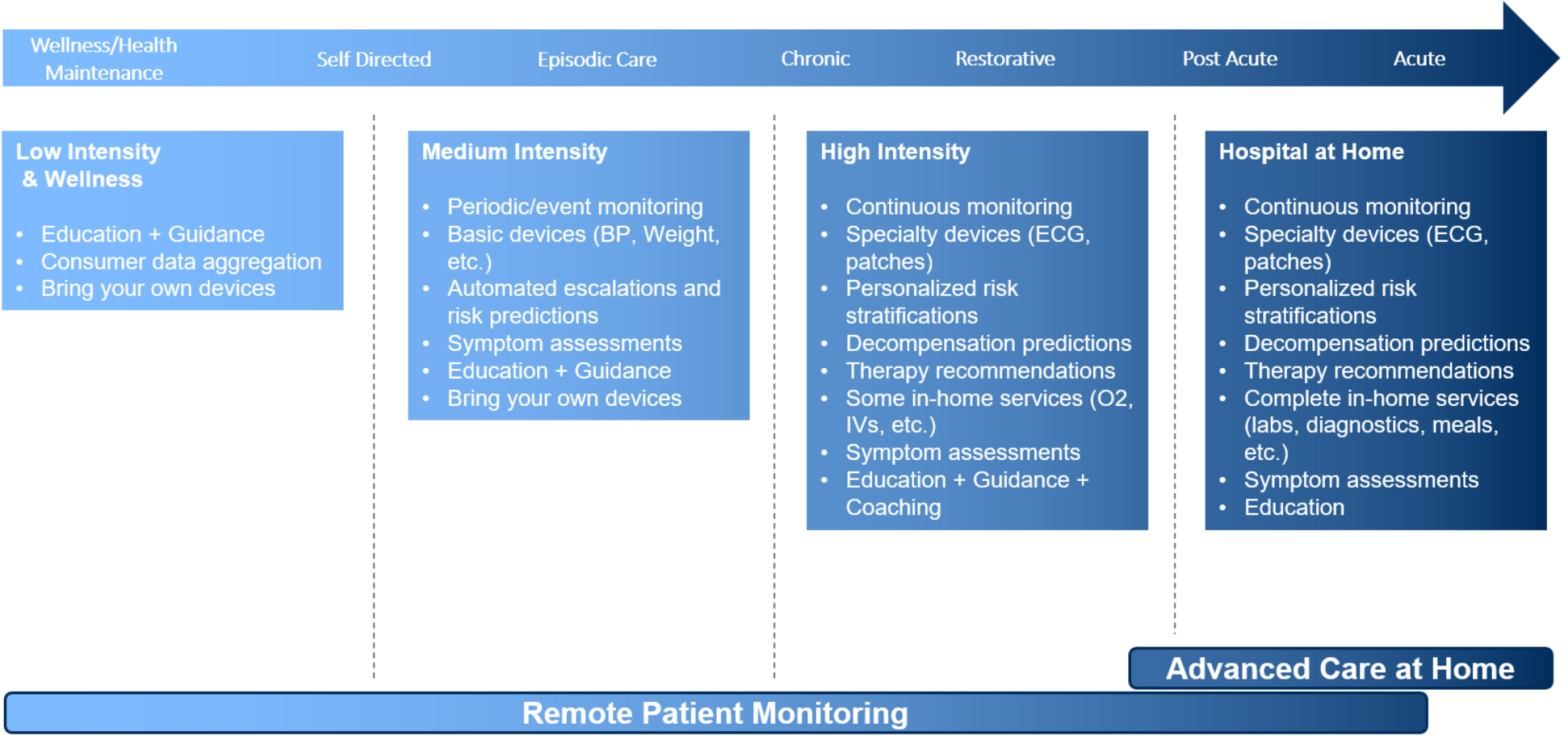
Matching RPM program components with patient needs.

The solution is designed to be vendor agnostic as well as adaptable to a variety of clinical situations. This adaptability increases the range of peripheral devices, wearables, and apps that can be integrated with the software platform and smart device. Similarly, this allows for adaptability based on individual patient needs, conditions, and acuity level, the range of the technology intervention (Figure 1).

The following factors were considered to maximize patient acceptance as the sophistication of the technology increased: implementing simple, user-friendly technology to optimize the patient experience and confidence in operating the equipment; utilizing cellular service to transmit data, and expand program access to patients in rural areas or those without internet connectivity in their homes; avoiding technology solutions that could exacerbate inequities in digital health access and literacy; partnering with external vendors to leverage their expertise and infrastructure to support device procurement, patient onboarding, device activation, inventory management, and equipment delivery and retrieval; integration of data from vended RPM software into the EHR for patient care and regulatory purposes to optimize workflows.

### Clinical operational model

One of the most critical factors for a successful RPM program is a clinical care team to monitor data and respond to alerts efficiently and effectively (46). Medical management of patients includes, but is not limited to, medication adjustments, treatment plans, coaching and counseling, and care coordination. Various governance and staffing models were considered:

• **Centralized:** Monitoring is confined to a centralized group of RPM RNs, advanced practice providers (APPs), and physicians. Most commonly in this model, the staff is exclusively focused on providing the RPM service with no concurrent job responsibilities. This can facilitate broad scalability; however, staff may have limited specialty practice knowledge and experience.
• **De-Centralized:** Monitoring is spanned across multiple departments and health care providers each take responsibility for their patients' care. Most commonly these monitoring duties are a component of individual nurse's or provider's daily routines, and they are responsible for a smaller panel of patients limited to their personal practice, care team, or specialty. This can facilitate continuity of care, but it can limit scalability.
• **Hybrid:** Monitoring is conducted by a centralized team of RPM RNs who are responsible for the “first look” at the data, triaging the adverse data trends and alerts, and passing on the validated care escalation to the appropriate primary or specialty care APP or physician who assumes the patient's clinical oversight.

### Technology supply chain and support

To ensure the scalability of RPM programs, operational infrastructure and processes must be developed to support inventory management, maintenance, deployment, and recovery of monitoring equipment. This includes the packaging of RPM equipment across distribution methods such as direct-to-patient and the use of distribution hubs for local pick-up. The remote patient monitoring “kit” includes the remote monitoring equipment, packaging material to protect the equipment, and operating instructions. It is important to balance the use of a standard kit with specialty kits to support niche program needs and reduce complexity. This ensures scalability across multiple distribution methods. Once ready to ship, delivery services should be selected that offer flexible pick-up and delivery times. Following kit receipt, processes and services should be available to support installation and onboarding into the program.

As patients utilize their equipment, support should be available to assist with any technical issues that arise with the software or equipment. Support can include quick reference guides, video tutorials, chat, and phone support. Having a range of options ensures that patients have access to non-clinical, technical experts for support outside of the traditional clinic hours. Once a patient graduates, the RPM kit must be retrieved and returned for inspection, cleaning, and re-calibration of devices.

While enrolled in the RPM program, the technology and support structures should be utilized to promote patient engagement and increase adherence to program requirements, such as daily vital sign and symptom reporting. The technology should be leveraged to follow up with patients when they miss their daily reporting tasks. For example, the monitoring software could automatically notify patients through the device that they have missed their testing time. This could be combined with manual processes, such as non-clinical staff placing outbound calls to patients who have not yet completed their testing.

Successful RPM program operation requires the support team to have visibility across all aspects of the supply chain to monitor service levels. This includes the ability to track and monitor kit pick-up, delivery and retrieval times, verification of patient onboarding, monitoring of support volumes and wait times, and kit turnover rates. Having access to these data enables the product operations team to monitor the quality of processes and to measure the impact of any quality improvements implemented. This can translate to enhanced patient satisfaction and care team confidence that patients will receive their kit timely, be onboarded quickly, and participate fully in the program.

### Legal and policy

There are several legal and policy considerations incumbent in developing an RPM program. It is important to define the appropriate patient profile and selection criteria to ensure patients fit with the RPM service and match the clinical staffing competencies. While clinically beneficial for chronic condition management, RPM does not offer the same type of continuous monitoring as an electronic intensive care unit (eICU) or other higher acuity setting. With clinical criteria and protocols in place, it is important to consider the regulatory environment of RPM service implementation within a clinical practice and to proactively build compliance with federal and state standards into the program by design. Common considerations include scope of practice and professional licensure requirements at the state level, as well as billing policy which may be regulated at either the federal or state level depending on the payer(s) of interest.

#### Licensure & scope of practice considerations

The licensing of healthcare professionals falls within the regulatory authority of each state. Licensing Board structures may vary from state to state. However, each type of licensed professional, generally, has its own licensing board charged with overseeing the standard of care, the appropriate scope of practice, and disciplinary consequences of the individual licensees under the board's purview.

The provision of RPM-type care is ubiquitously agreed upon by both state boards of medicine and nursing as a type of clinical interaction which would constitute the practice of medicine and/or the practice of nursing; therefore, RPM would fall under the regulatory enforcement of the corresponding state board and require a license for a healthcare professional to engage in that activity within that state (47). A license is required to provide RPM services, and professionals must comply with the relevant standard of care owed to the patient, reiterating the importance of clearly articulated care protocols and patient inclusion criteria upfront to match patient needs with the right level of care. Since RPM is often a nursing-led modality of care, the RPM RNs must also act within their allowed scope of practice and have the appropriate training, education, experience, competency, as well as appropriate level of supervisory oversight. At the functional level, the tasks a nurse performs during an RPM encounter are not altogether different from those a nurse might perform within a hospital or clinic setting. Most state licensing boards have not yet distinguished between “in-person” and “digital” scopes of practice for intrastate activity, though supervision requirements for clinical staff and/or required competencies may vary between states (47).

The provision of clinical care across state lines increases licensure complexity. Providers caring for patients *via* remote monitoring will likely need licenses in both the state where they are located and where the patient is located at the time of the service. Additionally, providers must adhere to the applicable scopes of practice standards for both states. Deployment of an RPM program requires a careful review of licensing requirements and state regulations for each state where potential participants reside.

Multiple types of legislative vehicles exist to increase licensure portability across state lines and can reduce some administrative burden to providers when pursuing new licenses. Licensure compacts provide healthcare professionals with an expedited path to a new license in another state that has also enacted the compact. There are licensure compact options available for multiple types of providers, and their popularity has grown in response to the COVID-19 pandemic. Multiple states have enacted agreements like the Interstate Medical Licensure Compact for Physicians and Nurse Licensing Compact in recent years, and portability compacts agreements for new provider types (e.g., social workers and masters-level mental health counselors) are in development with possible enactment in 2023. Unfortunately, compacts are not one-size-fits-all, and details vary between provider types. Generally, compacts are not a reciprocal license that grant authorization to practice in the new state, rather they are an expedited avenue to a new license issued by a new medical board, and thus still entail separate fees and education credits, among other requirements.

Separately, from licensure compacts, some states have enacted telehealth registration pathways or “special purpose licenses” which allow out-of-state providers to register with the local medical or nursing board to see patients within that state *via* telemedicine. Where available, these pathways can be efficient means of gaining authorization to practice as an out-of-state provider (usually for a lesser fee than a full or compact license)—though, as of this writing, these types of registration structures are far from common across the nation. Depending on the key states where potential RPM participants reside, one or more of these legislation options may be available for providers, though as noted, each professional must still be mindful of the unique state-level standards of care and scope of practice requirements as their licensure portfolio expands.

#### Billing & reimbursement considerations

Given the variety of payers common within the modern healthcare ecosystem, compliant billing requires analysis and vetting of both federal and state law. Regardless of the payer, the RPM service is reflected in a set of four Clinical Procedure Terminology (CPT) codes as defined by the American Medical Association. In brief, they are:

• CPT 99453: an initial setup code reflecting patient education on use of their remote monitoring devices
• CPT 99454: a code reflecting the transmission of initial patient parameters
• CPT 99457: a code reflecting 20 min of interactive communication time between patient and provider during a calendar month period as part of the RPM service; and
• CPT 99458: a code for an additional interactive communication time between patient and provider, billed in 20-minute increments as appropriate to reflect the service rendered.

To appropriately bill any government or commercial payer for the RPM service, all elements of the code must be met. Additionally, documentation within the patient's medical record is required to justify the service performed. A significant portion of the service is represented by aggregated clinical interactive time across a calendar month; thus, the provider performing the service must document the duration of interactive time spent with the patient to justify the services rendered. Patients may be on similar services (e.g., chronic care management) that are also working toward a goal of empowering the patient to proactively manage their chronic care needs. If this is the case, it is crucial to carefully count time spent performing RPM separately from the time performing those parallel services that seem similar in nature. The time and documentation for each service should stand on its own, and consistent policies should be established, documented, and adhered to governing how time is allocated in billing for these codes.

The Centers for Medicare & Medicaid Services formalized the current incarnation of the RPM codes in the agency's Physicians Fee Schedule final rulemaking, published in 2018 (48). Except for a clarification the following year which made the codes eligible to be performed by ancillary clinical staff working under general (as opposed to direct) supervision of the billing provider, the required billing criteria have been relatively stable since the codes' inception (49). It is important to research the appropriate scope of practice for non-nursing professionals ahead of implementing (and ultimately billing for) an RPM program. As with all services reimbursed under the Medicare Part B program, Medicare beneficiaries will be assessed a copayment each time the RPM CPT code(s) are billed (48).

RPM has also been received favorably at the state level, with at least thirty states including RPM coverage in their medical assistance programs, though they may impose additional requirements on when the services are covered (50). Some states may also have provisions mandating coverage of remote monitoring services for commercial payers. However, many commercial payers may elect to do so for their covered beneficiaries even absent such mandates. Commercial coverage mandates for RPM are less common than similar legislative mandates for other modalities of virtual care (e.g., video telemedicine or even asynchronous connections), though private payers may elect to cover these services within their benefit plans (51). Similar to other healthcare services, patients may be responsible for any co-insurance, co-payments, or portions that are beyond what is covered by their insurance. For patients who demonstrate a need, healthcare institutions may consider providing financial assistance to cover these patient-responsible components.

## Methods

### RPM program implementation and goals

Mayo Clinic is a not-for-profit group practice with integrated research, education, and clinical practice activities with over 5 million outpatient visits annually across a multi-campus environment spanning five states (52, 53). In early 2015, remote monitoring offerings existed as isolated projects and research studies within the institution. These services were fragmented, non-standardized, nor scalable (54). To address this, an “Area of Excellence” with centralized program management was developed to establish clear governance and oversight, standardize clinical practice and reporting, and coordinate partner and vendor relationships (55).

The RPM program was established as a new care transitions and population health management model of care with goals and corresponding metrics by which program impact can be assessed (Table 1). The overall strategy for this service was to utilize RPM technology and nursing care coordination to drive patient engagement in disease or post-procedural management with the aim to improve health outcomes while concurrently reducing health care utilization. Furthermore, the program aims to directly engage patients in their health care and to graduate the patient to self-management, leading to better long-term outcomes.

**Table 1:** Overall RPM program goals and corresponding metrics.

RPM program goals^a^	Corresponding metrics
Improve patient experience and increase satisfaction	Patient satisfaction survey and scores
Improve patient health outcomes	Objective health measures (mean blood pressure and hemoglobin A1c, mortality, etcetc.)
Increase patient engagement	Patient activation questionnaire and scores
Reduce Emergency Department visits	Risk-adjusted number of emergency department visits
Reduce hospitalizations and inpatient observations	Risk-adjusted total hospital days
Shorten hospital length of stay^a^	Risk-adjusted initiating hospital length of stay^a^
Reduce hospital readmission rates	Risk-adjusted 30-, 60-, 90-day hospital readmission rates
Increase efficiency of care/Reduce outpatient care coordination burden	Number of EHR telephone encounters, patient portal messages, and provider inbox tasks
Extend geographic reach to rural communities	Geographic radius of RPM patient panels
Utilize care team resources to the highest level of license	Number of care coordination tasks completed by each care team member

^a^ For patients enrolled while in an inpatient status for post-hospital monitoring.

A centralized operational model, managed by a coordinating center, was chosen to streamline monitoring, coordinate investment of resources, and maintain staff's virtual care proficiencies. The RPM program utilized a matrix model wherein the operating budget resided within a centralized business unit even though some personnel are supervised by their home department. By establishing a dedicated monitoring staff, the RPM program avoided redundancy in staffing. In addition, the goal of developing a common data analytics infrastructure was to automate patient data collection and curation, facilitate quality reporting, and generate a repository for predictive modeling and research purposes.

Although many patients may benefit from RPM services, it may not be practical or scalable from a cost-benefit perspective to enroll all patients. Based on an analysis of hospital data, it was determined that select patient morbidities would provide a significant opportunity for a positive impact by the RPM program to reduce hospital admissions, readmissions, intensive care unit (ICU) days, and length of stay. Mayo Clinic developed a centralized nurse-led RPM program for the management of patients with complex chronic conditions (congestive heart failure, chronic obstructive pulmonary disease, uncontrolled diabetes, and uncontrolled hypertension) aimed to improve patient experience, clinical outcomes, and reduce health care utilization. This traditional RPM model of episodic assessment of subjective symptoms and objective physiologic data was implemented in the community-based, Mayo Clinic Health System sites in 2016 and subsequently expanded in 2018 to the tertiary centers in the Midwest (Rochester, Minnesota), Southeast (Jacksonville, Florida), and Southwest (Scottsdale, Arizona). In addition to site expansion, beginning in 2018, the RPM framework was leveraged to support new programs for patients following high-risk surgery/procedures and those with specialty conditions. Similarly, an RPM program for patients with acute COVID-19 at risk for severe disease was implemented at the onset of the pandemic in March 2020 (3, 56). As such, program length ranged from 14 to 90 days, with a shorter length for acute conditions and a longer duration for chronic condition management. Mayo Clinic subject matter experts in each of the disease management domains worked collaboratively with the RPM clinical nurse specialists and nurse manager to develop enterprise-endorsed order sets and decision trees that included patient eligibility criteria, medication titration tables, vital signs parameters, symptom assessments with alerting thresholds, and disease management for alerts.

### RPM program workflows

The general workflow of this RPM model involves a patient at home or remote from a Mayo Clinic facility who receives a technology package comprised of a tablet with cellular service and pre-connected, Bluetooth-enabled devices (blood pressure cuff and monitor, pulse oximeter, and scale). A thermometer and glucometer for self-reporting are also included if indicated. The tablet notifies patients to perform vital sign measurements and complete condition-specific symptom assessments once daily for chronic condition programs, or more frequently for acute care (COVID-19) and specialty care programs. The technology passively captures physiologic information and records self-reported symptom assessments. The PGHD are transmitted from the technology to a hub integrated within the EHR, where it is validated and tracked.

**Figure 2:**
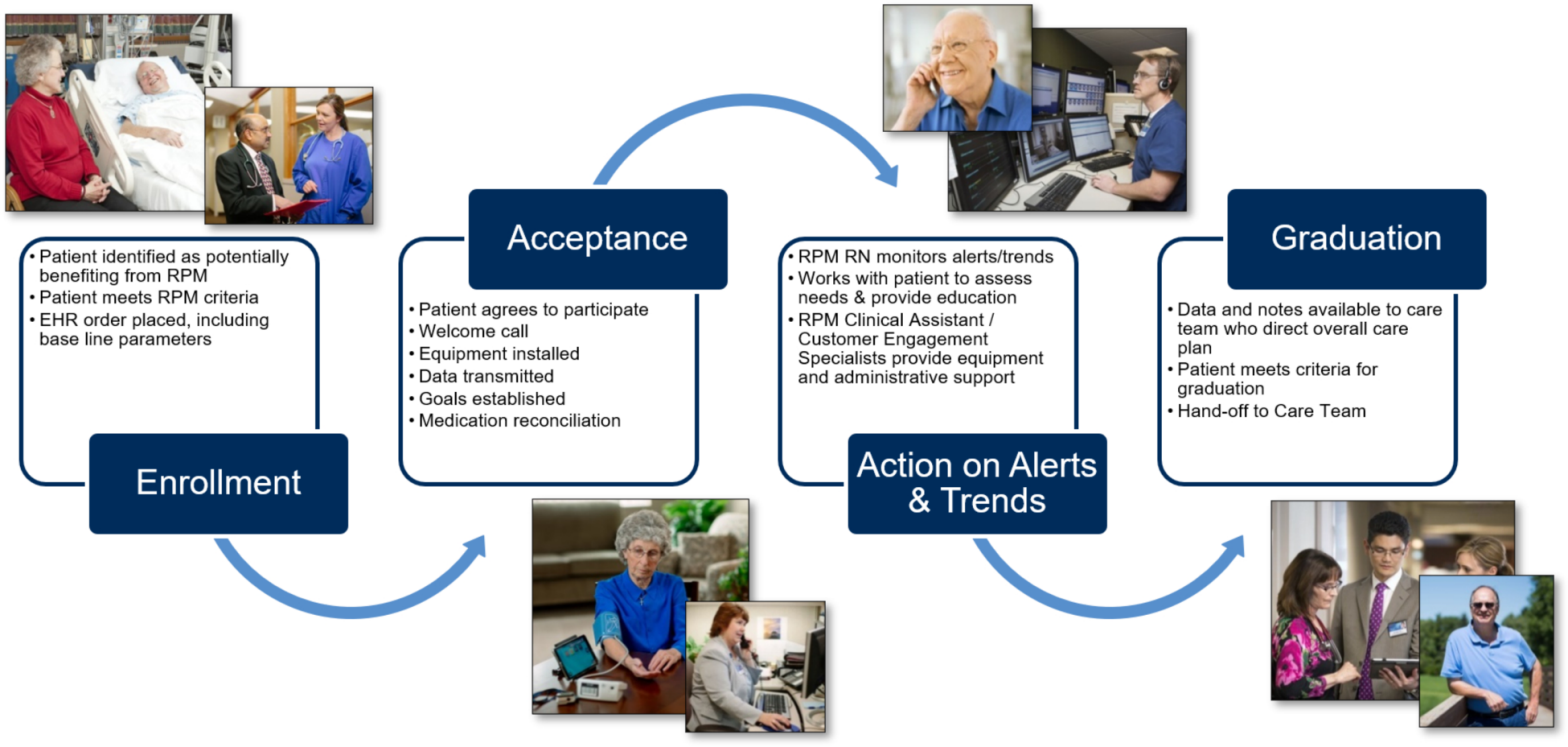
High-level patient flow through the remote patient monitoring program.

Clinical assistants (CAs) coordinate patient needs that do not require clinical decision-making skill sets, such as patient enrollment, technical support, PGHD validation for aberrant results, and patient adherence to RPM tasks. If the PGHD demands a response, an alert is passed to a centralized team of RPM RNs who, guided by standardized decision trees and protocols, use these EHR-integrated data to inform and provide individualized patient assessment, patient education, medication management, goal setting, and clinical care planning. When needed, they escalate care to the patient's primary or specialty provider. Documentation occurs within the EHR. The overall patient flow through the RPM program is summarized in Figure 2. The virtual, centralized team of RPM RNs support patients across all of Mayo Clinic's practice locations and is structured to support patients for 16 h per day, seven days a week. The multidisciplinary staffing plan was essential to developing standardized solutions and enabling the growth of RPM in care delivery.

### Patient identification, enrollment, and activation

In accordance with credentialing requirements and EHR access, a Mayo Clinic-affiliated managing provider was required for patient eligibility and participation. Most often, the patient's primary care provider (PCP) served as this managing provider, although under certain programs this may be a specialty provider (e.g., Cardiology, Surgery, Oncology, etc.).

Patients are identified, enrolled, and activated to the RPM program through a multi-step process. High-risk patients are identified by the RPM RNs at or following hospital discharge. Predictive scoring models were successfully deployed to aid in patient identification for RPM including the Elder Risk Assessment model (57), which uses administrative data to identify patients at high risk of readmissions or emergency department visit, and the LACE index for hospitalized patients, which utilizes length of stay, acuity of the admission, comorbidities (measured with the Charlson comorbidity index), and previous emergency department visits before readmission, to calculate predicted risk of hospital readmissions and early death (58, 59). Identified patients are screened for program eligibility criteria (Table 2). RPM RNs contact all eligible patients by telephone within two business days of index hospitalization discharge. If the nurse is unsuccessful in reaching the patient on two attempts that will be considered “no contact.” An established post-hospital follow-up telephone call format is followed. During the phone call, the nurse will review post-hospital appointments and adjust timing based on patient status. The program was expanded to allow PCPs to refer patients with chronic conditions at risk for frequent emergency department visits and/or hospitalization (“rising risk”) to the RPM program from the ambulatory setting.

**Table 2:** General remote patient monitoring inclusion and exclusion criteria.

	Chronic & acute RPM programs^a^	COVID-19 RPM program
Inclusion Criteria	• Age 18+ • English Speaking or has individual in patient's home who is willing to assist the patient with written instructions and education • Mayo Clinic PCP or Specialty provider involved in managing care • Patient requires vital sign monitoring and/or condition/symptom assessment • Willing to actively use technology	• Age 18+ • Patients at high risk for severe COVID-19 illness were eligible for the high-intensity care model if they had one or more of the following: age >65 years, diabetes, current smoker, BMI >40, chronic liver disease, chronic lung disease, congestive heart failure, active cancer therapy, bone marrow or solid organ transplant, other immunocompromised state, end-stage renal disease. Additionally, patients were eligible if they were hospitalized for COVID-19 without one of these risk factors but experienced one of the following: hospital length of stay ≥7 days, ICU admission, cardiac complications, need for mechanical ventilation or dialysis, need for oxygen supplementation at discharge, and receipt of remdesivir upon discharge. • Willing to actively use technology
Exclusion Criteria	• Patient is identified as end of life by provider • Resides in or is being discharged to a long-term care facility • Patient has dementia, cognitive impairment, (including recent suicide attempt/suicidal ideation or recent hospital admission for drug overdose), or physical condition that limits ability to use home remote monitoring equipment independently, or interact with remote patient monitoring staff (unless a caregiver commits to assisting daily)	• Patient is identified as end of life by provider • Resides in or is being discharged to a long-term care facility • Patient has dementia, cognitive impairment, (including recent suicide attempt/suicidal ideation or recent hospital admission for drug overdose), or physical condition that limits ability to use home remote monitoring equipment independently, or interact with remote patient monitoring staff (unless a caregiver commits to assisting daily)

^a^ Note: condition-specific criteria are applied on a program-by-program basis.

For patients who meet eligibility and express willingness to participate, the RPM RN arranges program ordering and vital sign goal setting with the patient's managing provider. An RPM CA contacts the patient to complete their enrollment and coordinates the delivery of the RPM equipment. The CA also sends printed and video program information (see Supplementary S1) and condition-specific educational materials to the patient.

RPM equipment is shipped overnight directly to the patient's home by a third-party logistics vendor contracted by Mayo Clinic or delivered the same day by Mayo Clinic's courier service. Upon receipt of the equipment, patients can self-activate using the quick start guide (see Supplementary S2) or wait to be contacted by the vendor. The customer engagement team contacts the patient to ensure the patient has successfully activated their equipment, that vital signs are flowing into the monitoring hub, and that the patients' technology questions have been sufficiently answered. Once activation is completed, patients can begin monitoring.

### Nurse-led patient monitoring

The active monitoring stage of the RPM program includes daily monitoring of vital signs and symptom assessments, RPM RNs' evaluation of patients with adverse data trends and alerts, and follow-up every two weeks for patient self-management assessment, education, and tracking progress on established goals. RPM program participation is encouraged until the patient meets their individual program goals with an average program length of 90 days.

#### Daily vital signs monitoring and symptom assessment questions

Daily vital signs are recorded, transmitted, analyzed, and made available to support decision-making by the RPM RNs and care teams. RPM patients receive automated daily audible reminders to complete their vitals at a time that was specified during enrollment. Collected vital signs include weight, non-invasive blood pressure, heart rate, and blood oxygen saturation. These vital signs are collected *via* FDA class-two devices and transmitted to a tablet *via* Bluetooth connection. Depending on the patient's condition and needs, the patient can manually enter blood glucose and temperature (a non-Bluetooth thermometer is included with the kit). Additionally, patients are prompted to answer daily condition-specific questions *via* the tablet. All vital signs and questions are automatically transmitted to central management software and programmatically analyzed against condition-specific parameters for out-of-range readings and trends. Readings are automatically color-coded and sorted based on severity. Vital sign data is automatically transmitted to the EHR, where they are visible to the care team.

#### Management by RPM clinical assistants

RPM CAs interact with patients throughout the RPM program. Daily, they monitor all patients' daily transmissions for patient compliance and technology issues, calling any patient who either does not test within an hour of the scheduled time or who has an apparent issue. Additionally, they field any phone calls from patients, including general program inquiries, clinical questions, technical support questions, and administrative program needs. Clinical and program questions are addressed directly by the CA team or escalated to the patient's RPM RN as appropriate. Similarly, technical support needs are addressed as able or escalated to the technology distribution vendor for higher-level technical support. Administrative program needs are addressed directly by the RPM CA team. Typical administrative needs include pausing a patient because they are going on vacation, have been hospitalized, or are being admitted to a skilled nursing facility; changing patient testing times; and updating patient contact and demographic information.

#### Management by RPM nurses

The Mayo Clinic RPM program's nursing framework includes aspects from both the Naylor (60) and Care Transitions Intervention (61) models, including patient education and goal setting, medication management, and coordination of post-discharge services. It aims to empower patients to better self-manage their condition and further includes monitoring symptoms and progress with adjustment of the care plan as needed. During the initial patient assessment, RPM RNs complete medication reconciliation and condition-specific assessments. Patients further receive education on red flags, review of post-hospital self-care, goal setting, review of follow-up appointments, how to contact RPM RNs and emergency care instructions.

RPM RNs contact program patients based on reading/trend/question responses and alerts. Following initial RPM RN assessment, trends or alerts of concern that need provider input are communicated to the managing provider *via* EHR messaging. The RPM RNs facilitate direct communication between the patient and managing provider for urgent issues. Patients are encouraged to communicate directly with the managing provider for other routine/non-urgent issues unrelated to the RPM program.

The RPM RNs contact the patient every two weeks to review established goals, provide ongoing self-management education, review the plan of care, and discuss challenges/issues the patient identifies. RPM RNs collect baseline patient data and various measures used as part of the care transition. In addition, if deemed necessary by the RPM RN, patients complete the two-item Patient Health Questionnaire (PHQ-2) and two-item General Anxiety Disorder questionnaire (GAD-2). Patients screening positive on these tests complete the nine-item Patient Health Questionnaire (PHQ-9) (62) and/or seven-item General Anxiety Disorder questionnaire GAD-7 (63). Patients also complete the Patient Activation Measure questionnaire (PAM) (64) at baseline, 30-, 90- and 180-days, as well as receive a patient experience survey after graduating from the program.

### Patient graduation and disenrollment

As patients approach the RPM program duration or reach their individual program goals, they are considered for graduation. Prior to graduation, the RPM RN will review the patient's progress in areas of knowledge, skills/behaviors, status, and activation; verify the patient's vital signs have been stable; and confirm no recent medication adjustments within the past two weeks. Upon meeting these criteria, the RPM RN will recommend to the patient and their managing provider that they graduate from the program. Upon graduation, the patient will be transitioned through a warm handoff to the primary care team. Logistical steps are taken to coordinate the return of the equipment. including coordinated pickup of the equipment by a commercial shipping carrier. All returned devices are evaluated, cleaned (physically and digitally), standardized, and returned to inventory.

A proportion of patients opt out of the RPM program at some point between patient enrollment and planned graduation. Attempts are made to encourage continued participation and to understand and record all requests for canceled enrollments or program drop-outs.

Understanding patients' experience and level of satisfaction with the program is essential to ensuring high value care. As the patient leaves the program (whether they graduate or disenroll) they receive an email survey with questions relative to their comfort with the program, experience with the equipment, perceptions of communication, and overall experience (see Supplementary S3). Questions are primarily assessed on a five-point Likert scale – (1) strongly agree; (2) agree; (3) neither agree nor disagree; (4) disagree; (5) strongly disagree – and are accompanied by two open–ended questions.

### General operating model

The multidisciplinary Remote Patient Monitoring Oversight Committee was established to provide direction and oversight to RPM product development and implementation within the clinical practice. This committee is accountable for ensuring the alignment of RPM goals and priorities with the overall Mayo Clinic telehealth and virtual care strategy. It is charged with (1) developing and overseeing service line business planning; (2) developing and overseeing scalable operational strategies and tactics; (3) partnering with administrative leaders and clinical practice stakeholders to review customer needs and align those needs with available or new service line offerings; (4) prioritizing and sponsoring projects to design/develop, implement, and improve RPM services; and (5) monitoring and ensuring stable, scalable and diffusible technologies and services to support clinical practice and patient needs. The integrated RPM service line is responsible for the following:

• **Product Management:** Understands the voice of the customer (practice and patient) is essential to new product development and optimization. Ongoing market research is conducted to understand emerging care delivery trends and how others deliver products and services. Business case development based on customer input and research is needed to deliver the best products and services to the practice. Product lifecycle management is also pursued.
• **Operations:** Establishes consistent policies, procedures, and guidelines to ensure operational efficiency and adherence to legal, regulatory, and reimbursement requirements. Further, the team diffuses its standards and best practices as well as establishes metrics, quality monitoring, and analytics to ensure system reliability and responsiveness for patients and providers. Customer support provides ongoing relations with the practice and coordination of service delivery.
• **Technology:** Develops processes for RPM device evaluation and acquisition. The team provides systems for equipment calibration, shipping to patients, field support, and pickup when the RPM program ends. Equipment management services can be outsourced as these functions are not typical services offered by healthcare organizations. Reliable patient and customer technical support systems are established.
• **Knowledge Management and Quality Improvement:** Develops and maintains evidence-based, best practice protocols in collaboration with condition or procedure-specific subject matter experts for patient and condition management, to include alert criteria, documentation standards, decision trees, order sets and provider communication protocols. In addition to standard patient adherence criteria, satisfaction, and health outcomes metrics, this team defines condition and procedure-specific metrics for program monitoring and quality assurance.
• **Communications:** Develops communication materials for patients, caregivers, and the practice to explain the RPM program, its value proposition, and operational model to encourage patient participation and practice adoption.
• **Finance/Legal:** Provides expertise to inform program development and maintenance through consultation with Revenue Cycle, Contracting and Payer Relations, as well as Risk Management and Legal.

The benefits derived from a cross-departmental and multi-site, enterprise model include centralized (1) reporting and analytics, (2) knowledge and technology management infrastructure, (3) clinical operational unit, and (4) third-party vendor and partner management.

## Results

Since the inception of the RPM program, nearly 22,000 patients have been supported (Table 3). Four RPM programs for chronic condition management (congestive heart failure, chronic obstructive pulmonary disease, and hypertension) were implemented upon initial deployment in 2016. Six additional programs were implemented between 2018 and the first quarter of 2020 (coronary artery disease, type 2 diabetes, post-coronary artery bypass graft (CABG), post-acute myocardial infarction/percutaneous intervention (AMI/PCI), post-ICU, and post-chimeric androgen receptor T-Cell (CAR-T) therapy). In March 2020, the COVID-19 RPM program was implemented, during which time the patient census for other established programs was reduced to enable flexible capacity for anticipated viral surges throughout the multi-regional practice. After reactivating general RPM program development in 2021, six additional programs were implemented (cirrhosis, post-thoracic surgery, post-procedural, acute kidney injury, asthma, and neutropenic fever). In total, the RPM program has supported over 4,500 patients with chronic conditions, nearly 17,000 patients with COVID-19, and 550 patients following acute conditions, surgery, or procedures.

**Table 3:** Patients monitored per program, from RPM inception (2016) through September 2022.

Condition monitored	Year implemented	Patients supported to date
Congestive Heart Failure	2016	2,089
General Complex Care	2016	178
Chronic Obstructive Pulmonary Disease	2016	422
Hypertension	2016	1,470
Coronary Artery Disease	2018	204
Type 2 Diabetes	2018	29
Post-Coronary Artery Bypass Graft (CABG)	2019	203
Post-Acute Myocardial Infarction/Percutaneous Intervention (AMI/PCI)	2019	10
Post-Intensive Care Unit (ICU)	2020	31
Post-Chimeric Androgen Receptor T-Cell (CAR-T) Therapy	2020	114
Coronavirus Disease 2019 (COVID-19)	2020	16,872
Cirrhosis	2021	110
Post-Thoracic Surgery	2021	142
Post-Procedural	2021	5
Acute Kidney Injury	2021	36
Asthma	2021	1
Neutropenic Fever	2022	9
Other	N/A	27
Total		21,915

Table 4 summarizes detailed patient demographics and characteristics. Among the patients served through the various programs, the median patient age was 66 years. There was a near-even distribution of women and men, with the majority married or in a life partnership (64.5%). The overall population was predominantly white (89.3%), with Hispanic and Latino patients comprising 7.58%. Approximately 5.5% of patients spoke a language other than English as their primary language. While patients were enrolled across all three tertiary Mayo Clinic campuses or four MCHS regions in the Midwest, 14.4% enrolled from the community-based MCHS sites.

**Table 4:** Characteristics among patients monitored *via* RPM, from inception (2016) through September 2022.

Characteristic	
Age, years	
Mean	64.12
Median	66.25
Age Group	
0–17	3 (0.01%)
18–24	302 (1.38%)
25–34	1,125 (5.13%)
35–44	1,805 (8.24%)
45–54	2,496 (11.39%)
55–64	4,345 (19.83%)
65–74	5,806 (26.49%)
75–84	4,214 (19.23%)
85+	1,819 (8.30%)
Sex	
Female	11,094 (50.62%)
Male	10,821 (49.38%)
Marital Status	
Married/Life Partnership	14,127 (64.46%)
Single	3,290 (15.01%)
Widowed	2,187 (9.98%)
Divorced/Separated	2,144 (9.78%)
Unknown	167 (0.76%)
Race	
White	19,608 (89.47%)
Black or African American	858 (3.92%)
Asian	423 (1.93%)
Other	657 (3.00%)
Unknown	369 (1.68%)
Ethnicity	
Not Hispanic or Latino	19,859 (90.62%)
Hispanic or Latino	1,619 (7.39%)
Unknown	437 (1.99%)
Language	
English	20,702 (94.46%)
Spanish	760 (3.47%)
Other	418 (1.91%)
Unknown	35 (0.16%)
Empaneled to a Mayo Clinic Primary Care Provider	
Yes	19,723 (90.00%)
No	2,192 (10.00%)
Enrollment Setting and Location	
*Tertiary Sites*	
Rochester, MN	14,269 (65.11%)
Scottsdale/Phoenix, AZ	2,898 (13.22%)
Jacksonville, FL	1,503 (6.86%)
*Community-based Health System Regionals Sites*	
Southwest Minnesota	1,075 (4.91%)
Southwest Wisconsin	1,059 (4.83%)
Northwest Wisconsin	601 (2.74%)
Southeast Minnesota	409 (1.87%)
Unknown	101 (0.46%)

Patient satisfaction is utilized in healthcare as a measure of clinical quality, with ties to reimbursement from the Centers for Medicare and Medicaid Services (65). Mayo Clinic routinely captures patient experience data for all modalities of clinical care to measure patients' perceptions of the care they receive (66). Ninety-five percent of patients reported being satisfied with the RPM program and that they are likely to recommend RPM to a friend or family member. Eighty-three percent of providers reported overall satisfaction with the program, with eighty-eight percent likely to recommend future patients. In the COVID-19 RPM program, patient engagement rates of approximately 78% were observed (3). Ease in using the technology was also assessed, with 93% of patients reporting that the medical equipment was easy to use. Related to this, 89% of patients reported that they thought the team explained how to use the equipment and 94% felt comfortable interacting with the care RPM team by phone or tablet.

Outcomes associated with RPM reported in the literature have been mixed, with most strategies having been shown to positively impact patient management (44). Prior evaluations at Mayo Clinic have found that the use of RPM for supportive care for patients’ acute and chronic conditions has been associated with positive trends in acute care utilization, clinical outcomes, and total cost of care (56, 67, 68). In a retrospective analysis of patients who engaged in the COVID-19 RPM program, we observed a significantly lower rate of 30-day, all-cause hospitalization (13.7% vs. 18.0%, *P *= 0.01), prolonged hospitalization >7 days (3.5% vs. 6.7%, *P *= 0.001), ICU admission (2.3% vs. 4.2%, *P *= 0.01), and mortality (0.5% vs. 1.7%, *P *= 0.01) when compared with those patients who were enrolled to the program but did not engage with the RPM technology (56). Further retrospective evaluation found that cancer patients with COVID-19 managed with RPM had a significantly lower hospital admission rate (2.8% vs. 13%, *P *= 0.002) compared with those managed without the program (68). Similar results were observed in a prospective, randomized, controlled trial of patients with chronic conditions who were monitored with or without RPM upon hospital discharge. In this study, there was a significant reduction in 30-day readmissions among those supported by the RPM program (18.2% vs. 23.7%, *P *= 0.03) (67).

## Discussion

### Building upon lessons learned

There have been many lessons learned through our experience, and we consider the most valued aspects of our model to be the flexible, well-defined care team models which allow efficiency and expertise in virtual monitoring while maintaining clinical expertise within the partner unit; use of RPM for acute and post-acute patients which facilitates hospital decompression; use of objective data and advanced analytics to drive operational decision-making; and the focus on clear outcome measures to drive ongoing program design, conduct, and expansion.

The next generation of RPM programs will likely evolve with the emerging wearables and sensors that can provide continuous, around-the-clock patient assessment and a wider range of physiologic variables (45, 69, 70). As learned during the COVID-19 pandemic, traditional episodic RPM models are limited in scalability by virtue of the magnitude of data generated that requires manual monitoring of alerts and trends (45). Continuous monitoring devices commonly include data platforms with complex analytics and FDA-approved algorithms for alerts to adverse data trends (45, 70). Machine learning techniques can also be applied to adjust monitoring parameters on an individual and programmatic basis.

#### Flexible, well-defined care team models

Key to the RPM model is a virtual, centralized team of RPM RNs. While the RPM program was initially developed as a 90-day program for chronic condition management or a 30-day program for select post-surgical/procedural support, as new and more specialized RPM use cases have emerged, there was an identified need to include specialty nursing teams to address complex patient care. Redesigning the RPM care team model consisted of building purposeful workflow back to the specialty care teams, as indicated by the co-created defined parameters and workflow. The goal is to keep the RPM nursing specialty as virtual care and escalate care as appropriate to the partnering care teams for personalized care. This approach also aims to achieve efficient RPM staffing strategies and nurse/patient ratios while providing specialized care to patients.

Pilots are underway at our organization using new virtual care nursing models to provide acute post-hospital care. Further efforts are in progress to connect patients across their care continuum, as complexity and acuity change with time, to provide a seamless transition between virtual care programs and primary and specialty care services. These models help ensure that virtual care expertise can be leveraged among highly trained nurses, after-hours staffing is available when needed, frontline staff can remain focused on their primary functions, and the host department's clinical expertise can be collaboratively leveraged rather than replicated.

#### Ambulatory and post-hospital care for acutely ill patients

At the onset of the COVID-19 pandemic, there was an urgent need to develop new ways to support ambulatory patients at risk for severe COVID-19 illness, decompress hospitals and EDs, and preserve personal protective equipment. With its technology, operational infrastructure, and clinical resources, the RPM framework was leveraged and rapidly scaled in response to the pandemic in support of patients with COVID-19 and underlying serious and complex conditions (3, 56). The RPM team built upon the experience and learnings from managing this acute patient population and partnered with the Department of Medicine-led “Remote Monitoring to Enhance Timely Hospital Dismissal” (REMODi) initiative. Through REMODi, the teams are jointly developing a scalable strategy to provide hospitalized patients with RPM, allowing for the safe yet rapid transition of patients to the ambulatory setting.

New models are being developed to provide hospital-level care using RPM technology and operational infrastructure. One such model being piloted allows patients to be discharged to a hotel with RPM following gastrointestinal procedures. This aims to replace the 24–48 h inpatient observation period, freeing up physical clinical space and reducing patient costs while maintaining a high level of patient care. Another model—termed Advanced Care at Home (ACH)—is implemented at several Mayo Clinic sites to bring monitoring, as well as diagnostic, therapeutic, and supportive care interventions into the patient's home, for management of higher acuity conditions and “hospital at home” care delivery (71–73).

Both RPM and ACH share the goal of providing high-quality care to patients outside of the hospital utilizing technology and a virtual care nursing team. Throughout 2021, ACH and RPM teams came together in iterative stages, with the end goal of complete collaboration between programs and the ability to seamlessly transition patients as their needs change over time. Stages in this collaboration include (1) seamless transfer patients to RPM following ACH hospital discharge to the restorative phase of the ACH program, (2) “admission” of an RPM patient to ACH when more intensive clinical needs emerge (e.g., in-home nursing and physician oversight, comprehensive medication management, infusion therapy, nutrition and rehabilitation services, etc.), and (3) expansion of APP and physician provider oversight to ensure access to RPM for patients who do not have a Mayo Clinic primary care or managing provider or to streamline complex care models with centralized APP and physician support.

#### Focus on outcomes to drive program decision making

**Figure 3:**
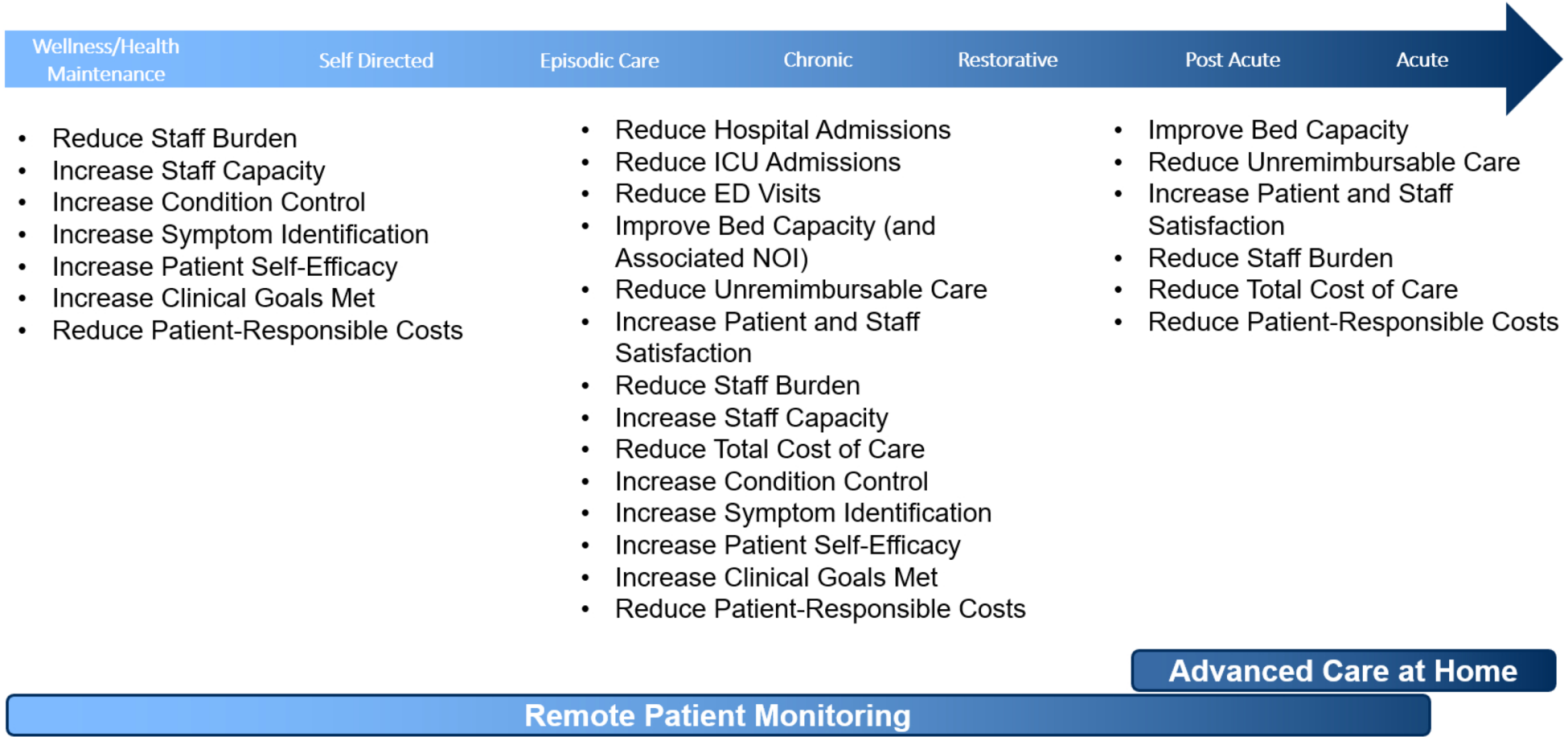
Value opportunities at different stages across the spectrum.

Utilization and clinical outcomes have been key drivers for program design decisions (Figure 3). These metrics have influenced patient and technology selection, care team models, and prioritization of clinical practice partners for continued program expansion. Formal health services research and quality assessments of RPM program outcomes are imperative for program evolution and necessary to drive needed policy and reimbursement reform (3, 56, 67, 68, 74). Intentional development of success metrics and integration of these outcomes' definitions occur early in RPM program development with practice partners. This includes clear outcomes definitions, baseline measurements, and remeasurement plans. Operational approaches were developed to systematically develop comparison (control) populations, allowing reporting of utilization and clinical outcomes in direct comparison and context to a like population who did not receive RPM.

### Opportunities for RPM program enhancement

#### Digital health equity

To accommodate the identified target conditions and provide an adaptable enrollment process, inclusion and exclusion criteria were focused on patient morbidity or condition; physical and mental capacity; language restrictions; and suitable home environment and support systems (see Table 3). Although well intended, only “ideal” RPM candidates were eligible, and the program population served has been of limited racial/ethnic and socioeconomic diversity.

The development and utilization of digital health solutions for clinical care delivery must monitor and address digital health disparities amongst the patient populations they aim to serve (75). Thus, strategies to address disparities that impact patients' ability to access and utilize technologies will be required to achieve optimal and equitable clinical outcomes (3). Additional opportunities to extend access to more patients include making program instructions and education content available in more written and oral languages as well as ensuring the technology and interpreter services are available in multiple languages. At the time of enrollment, protocols can be developed to assess patients' technical readiness to participate in the program, and onboarding support can be tailored for specific patient communities to ensure they understand the RPM program value, how to use the technology, and how to access program support.

There is minimal research available that provides RPM program patient engagement rates that can be used for benchmarking. However, programs that implement tactics to address equitable access and use of digital health technologies can be expected to optimize engagement across diverse patient cohorts.

#### Enhanced analytics

As described within this manuscript, patient identification for RPM program enrollment has been based on using standardized patient risk scores (such as LACE+), aiming to estimate a patient's risk of readmission. This model was an evolution in the program, which originally relied on frontline teams to identify and refer eligible patients at the point of care. Similarly, alerts and patient care decisions have been based on parameterized measures and fixed decision trees. Although these approaches provided a reasonable starting point, they are limited in their ability to account for a patient's unique profile, risk factors, and clinical patterns.

In the digital age of healthcare, greater efficiencies and improved health outcomes at lower costs can be accelerated through the synergy of advanced data analytics with remote monitoring and clinician expertise. Integration of these technologies into the current practice with virtual care platforms allows for the development of solutions that provide easy, timely, and accurate access to patients' health data supporting better diagnostics, treatment, and supportive care. To fully optimize diagnoses and treatments, understand trends, predict health risks, and develop long-term care plans, whole-patient data is required. Despite the demonstrated usefulness of these PGHD, in the absence of a data analytics platform, it is not possible to harvest the full potential of these new technologies.

To address these current limitations, the RPM team has developed and validated machine learning models to identify patients for the RPM program and to predict their clinical trajectory while in the program. These models aim to better identify patients who may benefit from the RPM program rather than by their risk for utilization of acute care resources, and to earlier detect adverse data trends and intervene to reduce the risk of clinical decompensation. Many of the required data elements for these models are obtained from the EHR. Additionally, the team aims to facilitate clinical integration of these insights within the EHR as a clinical decision support tool with alert-delivery mechanisms to assist RPM nursing and care teams. Applications of artificial intelligence (AI) aim to bridge the gap in data integration and translation into actionable information by providing pragmatic insights and near real-time responses to vital patient characteristics.

These models have completed internal validation studies, and they have been integrated into the EHR as clinical decision support tools. They will undergo usability and acceptance evaluation and testing through a pragmatic clinical trial currently in development.

#### Enhanced technology platform

An overall philosophy for the RPM program was to establish models with associated technology, care team structures, engagement methods, and frontline support that serve patients across their clinical journey and the continuum of care. Oversight of the RPM program has evolved to focus on the value RPM will bring to the practice and identify and integrate new technology/approaches based on what that high-value opportunity requires. This has led to rapid identification and onboarding of new vendors and technologies, as well as the development of novel, innovative care team models. Additionally, this has meant working with shared service partners (e.g., information technology and contracting) to allow for a more agile and responsive technology environment.

**Figure 4:**
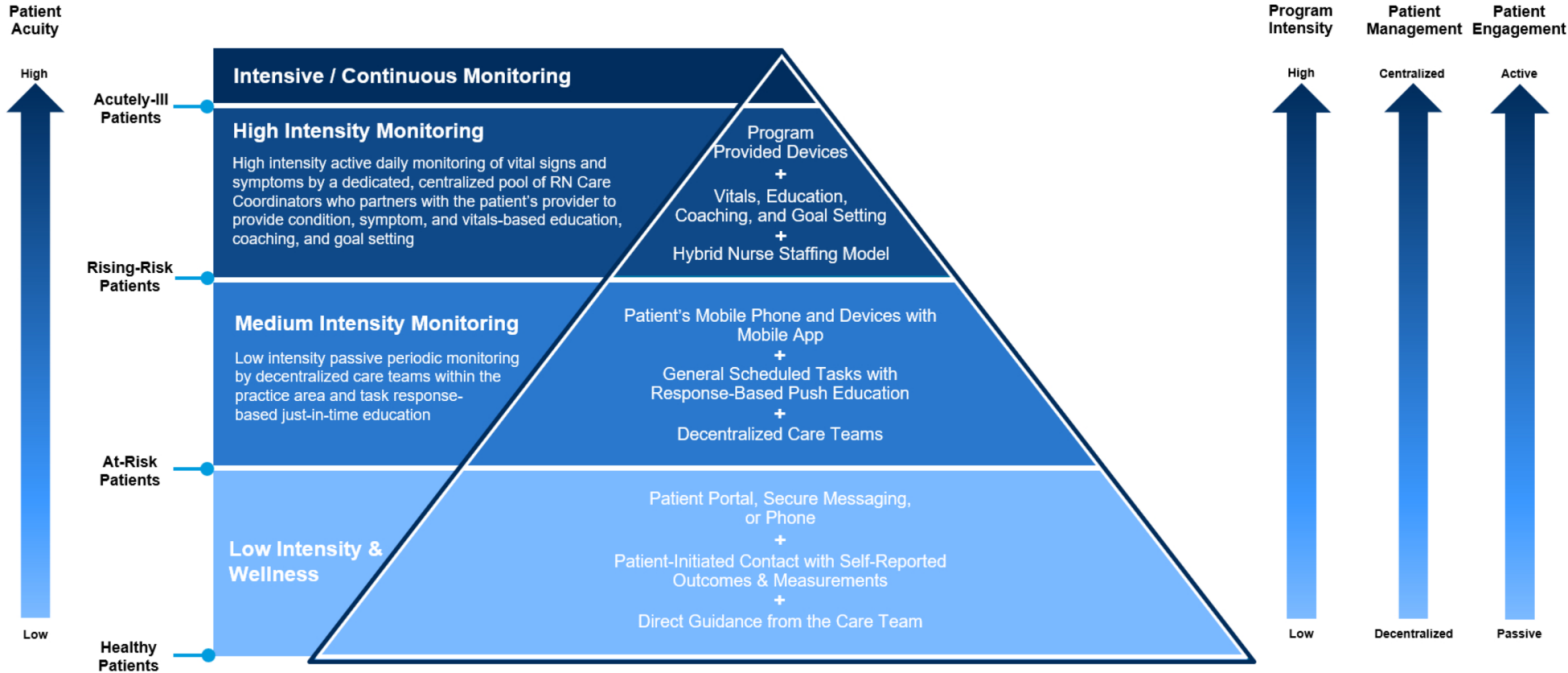
Framework for an RPM program with nurse-based care coordination.

To achieve the vision for the RPM program to offer a single solution that could easily adapt to meet the patient and care team needs as the patient's acuity and complexity evolve in their clinical journey, an adaptable technology platform that allows for the seamless addition/subtraction of integrated peripheral or wearable devices, education, communication modalities, and engagement methods was required to match patient and practice needs. Through a unified platform, care teams could customize the level of education and technology. Previously, this was achieved through an amalgamation of different technological solutions, each focused on independently meeting the needs of a particular segment of the patient population and stage within the clinical continuum (Figure 4).

Although these monitoring technologies were well suited to fit these specific tranches, they required a “rip and replace” of the technology and clinical workflows to adapt as patient needs changed resulting in a suboptimal patient experience. A new technology solution was identified that afforded a more adaptable and modular technology platform, with a focus on predictive analytics and device-agnostic architecture. Pilot implementation is underway to assess the performance of this new platform across the acuity spectrum—from programs targeting low-risk/low-acuity patients (i.e., those formally served through a separate low-intensity solution), moderate risk patients (i.e., those formally served by the RPM program), and high-risk patients (i.e., those who would otherwise be managed in the hospital). Additionally, pilots are planned to explore the management of patients as they change in their care needs, which would have historically required a transition between solutions.

#### Enhanced operational efficiencies

**Figure 5:**
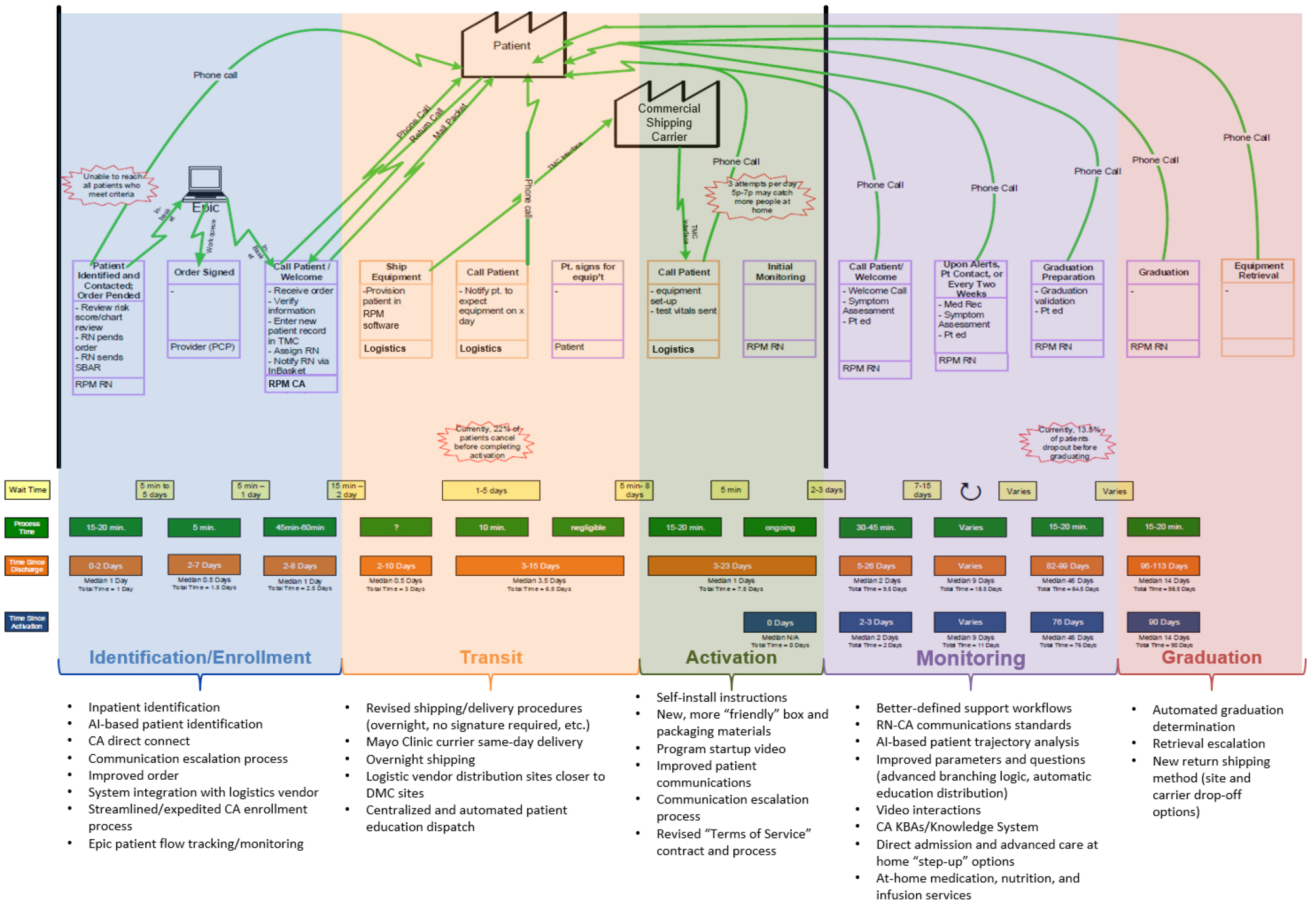
RPM program value stream map and quality improvement projects undertaken to improve efficiency.

Key to providing efficient and effective care under RPM is ensuring that the programs are backed by equally efficient operational models. The RPM team has undertaken quality improvement efforts to decrease the time for patients to receive equipment and become active in the program, improve patient access to resources and information, and maintain connectedness, while simultaneously decreasing program costs and staffing (74) (see Figure 5). Considerable improvements have been made in the logistics process, including more direct systems integration with our logistics partner to reduce human data entry and to implement new distribution processes. These include new multiple full-featured warehouse facilities distributed strategically relative to regional practices in the United States Midwest and Southwest to increase processing capacity. Additionally, new partnerships with local delivery partners allow direct, just-in-time distribution of kits to patients when shipping is not a timely option (74).

To reduce the effort per patient, new systems integrations and tools have been or are in process to be implemented, to allow more direct and efficient management of patient flows in our EHR, greater visibility into performance, and decreased needs for data entry and technical data management in downstream systems.

New partnerships have also been formed to facilitate the central processing and dispatch of physical education materials, reducing costs and the need to have staff on campus. Finally, patient and provider feedback are systematically gathered, aggregated, analyzed, and summarized using institutional processes and best practices through an active partnership with the Mayo Clinic Office of Patient Experience, with bi-directional interfaces allowing for automatic dispatches of surveys and direct integration of responses into the analytics platform.

## Conclusion

Chronic and acute disease management strategies are gaining greater focus as patient needs continue to rise. Key to achieving these strategies are more advanced methods of ambulatory care management, which provide for more rapid transitions from inpatient settings and positively impact overall care utilization. RPM has proven to be an effective way for Mayo Clinic to extend its care, effectively leveraging technology to connect patients and care teams and allowing for continual, early interventions which help avoid decompensation. The result has been a program that has positive clinical outcomes and is satisfying for patients and care teams alike. As technology advances, there are greater opportunities to enhance this clinical care model to provide more continuous and less disruptive means to capture patient-reported outcomes as well as to utilize more advanced technologies to enhance patient selection, trajectory analysis, and to guide interventions. This model can and should be extended and expanded to support patients across a broader spectrum of needs—from advanced, hospital-level services to ongoing supportive care. This report can serve as a framework for health care organizations to implement and enhance their RPM programs and provides some key areas for further evolution and exploration in developing RPM programs of the future.

## Data Availability

The original contributions presented in the study are included in the article/**Supplementary Material**, further inquiries can be directed to the corresponding author/s.
