# Supplementary material for: Development and implementation of a nurse-based remote patient monitoring program for ambulatory disease management
Source: Front Digit Health. 2022 Dec 14;4:1052408. doi: 10.3389/fdgth.2022.1052408 (PMC9794766; doi:10.3389/fdgth.2022.1052408)
Supplement: Supplementary file 2 [file Datasheet1.pdf]

MAYO CLINIC

Welcome to the

Remote Patient Monitoring Program

Quick Start User Guide

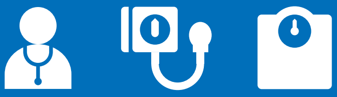

What's in the Box?

All or some of this equipment, depending on your specific needs.

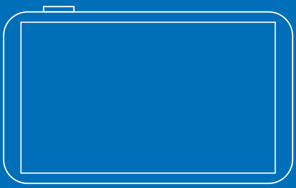

Tablet

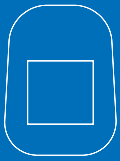

Pulse Oximeter

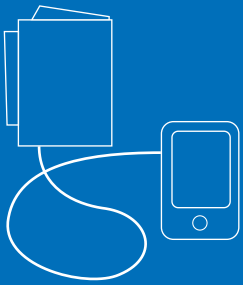

Blood Pressure Monitor and Cuff

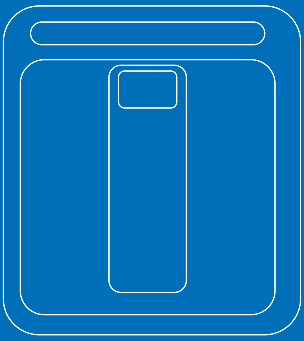

Scale

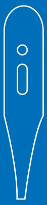

Thermometer

If you need medical assistance, please call the Remote Patient Monitoring care team

1-507-293-3371

For medical emergencies, dial

911

If you need help with your equipment, please call Equipment Support

1-800-910-7866

7 am-7 pm CT, Monday–Friday
8 am-4 pm CT, Saturday–Sunday

Quick Setup

1. **Find a good location for your remote patient monitoring equipment.**

2. **Unpack the equipment.**

3. **Turn on the tablet.**

   - Press and HOLD the power button on the top side of the tablet for 5 seconds.
   - Leave the tablet plugged in and turned on even when you're not using it.

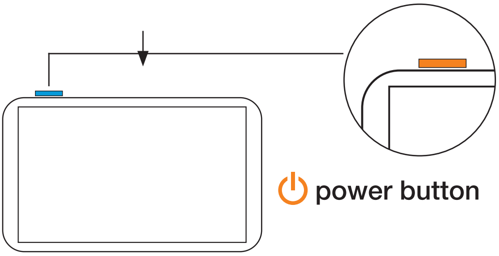

4. **Take and send your vitals to your care team.**

   - Sit down and relax for a few minutes.
   - Follow the on-screen instructions.

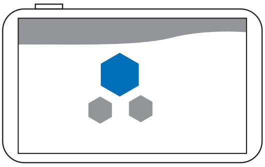

Tap **Vitals Now**

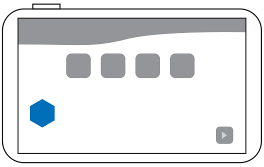

Then tap **Select All**

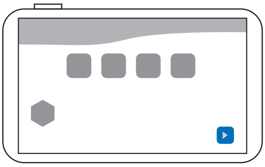

Then tap **Arrow** in lower right corner

   - Tap "Yes" to send your vitals to your care team.

Your vitals have been sent. Remember to take and send your vitals every day. In the next few days, someone from Mayo Clinic will call you to talk about your care plan.

How to Use the Pulse Oximeter to Measure Your Blood Oxygen

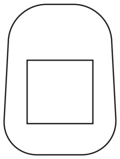

Pulse Oximeter

Preparing to measure your blood oxygen

- Sit quietly for 5-10 minutes before checking your blood oxygen to get the most accurate results.
- Make sure your hands are warm before you use the pulse oximeter.

Measuring your blood oxygen

1. Place the pulse oximeter's sensor (the part that opens and closes) on your index finger. Make sure your finger is completely in the sensor.
3. Place your hand on your leg, an armrest or some other non-moving surface.
4. Keep your finger straight.
5. Hold hand still until the tablet tells you to remove the pulse oximeter.

Once the pulse oximeter starts working, you should see regular blips on the pulse oximeter. These blips are marking your heart rate.

Helpful Tips

Dark fingernail polish or artificial nails may make it hard for the pulse oximeter to get an accurate reading. If the pulse oximeter is not working, remove the nail polish or artificial nails and try again.

If the reading seems inaccurate or is a very different reading than you normally see, try warming your hands. Rub your hands together or place them under warm water. Once your hands are warmed, put the device on another finger and follow steps 1–5 again.

How to use the Blood Pressure Monitor and Cuff

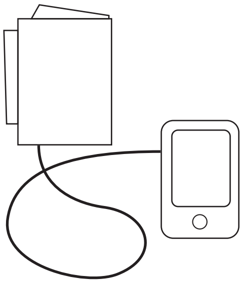

Blood Pressure Monitor and Cuff

Preparing to measure your blood pressure

- Take your blood pressure at about the same time every day, before 11 a.m. and before you eat breakfast.
- Do not smoke, eat or drink anything with caffeine 30 minutes before you measure your blood pressure.
- If you take blood pressure medicine, take your blood pressure one hour after taking your daily dose.
- Sit quietly for 5-10 minutes before taking your blood pressure to get the most accurate results.
- Make sure the air hose is attached to the monitor.
- Sit in a comfortable position near a table with your back supported, feet flat on the floor. Keep legs or ankles uncrossed.

Measuring your blood pressure

1. With your arm on a flat surface, palm facing up, place the blood pressure cuff on your bare upper arm. Make sure the air hose is on your arm reaching to the center of your palm. The cuff should be placed high enough so that one inch of your skin can be seen above the bend at your elbow.

   Refer to the illustration on the inside of the cuff for placement questions.
2. Tighten the cuff so that you can slide one finger between it and your arm.
3. Keeping your arm on the flat surface, make sure your arm bends at the elbow and the cuff is at heart level. Your arm should not be hanging by your side nor lifted towards your shoulder.
4. Push the start button located on the front of the monitor.
5. Remain still until the tablet tells you that you are done.

Helpful Tips

If you think the blood pressure reading is not accurate, wait 1 or 2 minutes. Check the cuff placement and retake your blood pressure.

How to Use the Scale

Preparing to use the scale

Use the scale in the morning, after you have emptied your bladder and before you eat breakfast.

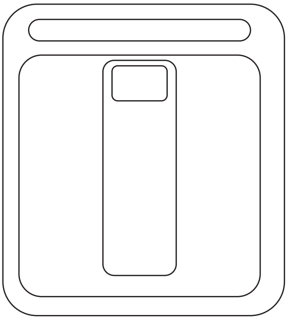

Scale

Using the scale

1. Stand very still, in the middle of the scale. Do not hold onto or touch anything around you. If you have difficulty doing this safely, please notify your Remote Patient Monitoring care team.
2. Remain on the scale until the tablet instructs you to get off.

Helpful Tips

Weigh yourself without clothing or wear the same type clothing (for example, similar shoes or slippers, clothing or pajamas of the same weight) each time you weigh yourself.

How to Use the Thermometer

Preparing to use the thermometer

- Sit quietly for a few minutes before using the thermometer to get the most accurate results.
- Avoid eating or drinking for about 15 minutes before you use the thermometer.

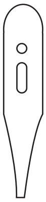

Thermometer

Using the thermometer

1. Place the thermometer under your tongue.
2. Remove the thermometer when you hear a beep.

Helpful Hints

Your Remote Patient Monitoring care team will let you know if taking your temperature is necessary.

Answers to Some Questions You Might Have

My tablet isn't working.

One reason your tablet may not be working is because it is no longer plugged in. Check to make sure that the power cord is plugged firmly into the port on the bottom of the tablet. Also make sure that the power cord is plugged into an outlet that is not controlled by a wall or light switch. If the tablet still does not work, call Equipment Support at 1-800-910-7866. A customer support representative will help you.

When I try measuring my vitals, I get error messages. What should I do?

Call Equipment Support at 1-800-910-7866. A customer support representative will help you troubleshoot the issue and confirm that your measurements are being sent to your care team.

What should I do if I am leaving my house for a few days while I'm in the program?

Remember that you can take your equipment with you if you will not be home for a while. However, if you prefer to leave the equipment at home, call your Remote Patient Monitoring care team at 507-293-3371 before you leave to let us know the dates you will not be home. Your care team will set your account as inactive for those dates. Please leave the tablet plugged in. When you return, start taking your measurements normally. If you return home early, call your Remote Patient Monitoring care team. The team will reactivate your account at that time.

MAYO CLINIC | 200 First Street SW | Rochester, MN 55905 | mayoclinic.org

©2022 Mayo Foundation for Medical Education and Research. All rights reserved. MAYO, MAYO CLINIC and the triple-shield Mayo logo are trademarks and service marks of MFMER.

MC0684-173rev0122
