# Supplementary material for: Development and implementation of a nurse-based remote patient monitoring program for ambulatory disease management
Source: Front Digit Health. 2022 Dec 14;4:1052408. doi: 10.3389/fdgth.2022.1052408 (PMC9794766; doi:10.3389/fdgth.2022.1052408)
Supplement: Supplementary file 3 [file Datasheet2.pdf]

Remote Patient Monitoring Patient Experience Survey

In an effort to gain a better understanding of patient needs and improving the Remote Patient Monitoring Program experience, we ask that you complete the following survey.

To begin the survey, please click on the "Start Survey" button below.

---

1. Please rate your level of agreement with the following statements about your comfort with the Remote Patient Monitoring Program.

	Strongly disagree	Disagree	Neither agree nor disagree	Agree	Strongly agree
The Remote Patient Monitoring Program helped me feel comfortable managing my health at home					

2. Please rate your level of agreement with the following statements about your equipment use in the Remote Patient Monitoring Program.

	Strongly disagree	Disagree	Neither agree nor disagree	Agree	Strongly agree
The team explained how to use the equipment					
The medical equipment was easy to use					
The equipment helped in my care at home					
I felt comfortable interacting with the team by phone or tablet					

3. Please rate your level of agreement with the following statements about communication with the team.

	Strongly disagree	Disagree	Neither agree nor disagree	Agree	Strongly agree
The team explained things to me in a way that was easy to understand					
The team listened to my concerns					
The team kept me informed about my care plan					
I was able to reach a member of the team right away for any questions or concerns					

The team promptly responded to my needs					
The team explained when I should seek medical attention					
The educational materials provided by the team were useful (i.e., information packets, booklets, pamphlets, etc.)					

4. Please rate your level of agreement with the following statements.

	Strongly disagree	Disagree	Neither agree nor disagree	Agree	Strongly agree
I felt ready to leave the Remote Patient Monitoring program					
The team treated me with courtesy and respect					
The staff worked well together to care for me					
I would recommend the Remote Patient Monitoring Program to others with a similar health condition(s)					
Overall, I am satisfied with the Remote Patient Monitoring Program					

5. Please tell us what **impressed you** about the Remote Patient Monitoring Program.

6. Please tell us what **disappointed you** about the Remote Patient Monitoring Program.
